# Effect of Folic Acid and Betaine Supplementation on Flow-Mediated Dilation: A Randomized, Controlled Study in Healthy Volunteers

**DOI:** 10.1371/journal.pctr.0010010

**Published:** 2006-06-09

**Authors:** Margreet R Olthof, Michiel L Bots, Martijn B Katan, Petra Verhoef

**Affiliations:** 1 Wageningen Centre for Food Sciences and Wageningen University, Wageningen, Netherlands; 2 Julius Center for Health Sciences and Primary Care, University Medical Center Utrecht, Utrecht, Netherlands

## Abstract

**Objectives:**

We investigated whether lowering of fasting homocysteine concentrations, either with folic acid or with betaine supplementation, differentially affects vascular function, a surrogate marker for risk of cardiovascular disease, in healthy volunteers. As yet, it remains uncertain whether a high concentration of homocysteine itself or whether a low folate status—its main determinant—is involved in the pathogenesis of cardiovascular disease. To shed light on this issue, we performed this study.

**Design:**

This was a randomized, placebo-controlled, double-blind, crossover study.

**Setting:**

The study was performed at Wageningen University in Wageningen, the Netherlands.

**Participants:**

Participants were 39 apparently healthy men and women, aged 50–70 y.

**Interventions:**

Participants ingested 0.8 mg/d of folic acid, 6 g/d of betaine, and placebo for 6 wk each, with 6-wk washout in between.

**Outcome Measures:**

At the end of each supplementation period, plasma homocysteine concentrations and flow-mediated dilation (FMD) of the brachial artery were measured in duplicate.

**Results:**

Folic acid supplementation lowered fasting homocysteine by 20% (−2.0 μmol/l, 95% confidence interval [CI]: −2.3; −1.6), and betaine supplementation lowered fasting plasma homocysteine by 12% (−1.2 μmol/l; −1.6; −0.8) relative to placebo. Mean (± SD) FMD after placebo supplementation was 2.8 (± 1.8) FMD%. Supplementation with betaine or folic acid did not affect FMD relative to placebo; differences relative to placebo were −0.4 FMD% (95%CI, −1.2; 0.4) and −0.1 FMD% (−0.9; 0.7), respectively.

**Conclusions:**

Folic acid and betaine supplementation both did not improve vascular function in healthy volunteers, despite evident homocysteine lowering. This is in agreement with other studies in healthy participants, the majority of which also fail to find improved vascular function upon folic acid treatment. However, homocysteine or folate might of course affect cardiovascular disease risk through other mechanisms.

## Editorial Commentary

**Background:** Evidence from observational studies indicates a link between high concentrations of homocysteine (an amino acid) in the blood and increased risk of cardiovascular disease. However, the basis for the link between homocysteine concentrations and cardiovascular disease risk is not clear. Supplementing the diet with B-vitamins lowers homocysteine levels, and large-scale trials are underway that will determine whether B-vitamin supplementation has an effect on cardiovascular outcomes, such as heart attacks and strokes. These trials also involve administration of folic acid as well as other B-vitamins. It is not obvious, however, whether the effects of B-vitamin supplementation arise as a result of homocysteine lowering or via some other biochemical pathway.

**What this trial shows:** Olthof and colleagues aimed to further understand the effects of homocysteine lowering by randomizing 40 healthy volunteer participants to receive either folic acid supplementation; placebo; or betaine, a nutrient that lowers homocysteine levels via a different biochemical pathway than folic acid. Each participant in the trial received each supplement for 6 wk, with a 6-wk washout period before the next supplement was given. The researchers then used a technique called flow-mediated dilation (FMD) to measure functioning of the main artery of the upper arm, as a surrogate for cardiovascular disease risk. In this trial, both folic acid and betaine supplementation significantly lowered homocysteine levels over the 6-wk supplementation period. However, both forms of supplementation failed to result in any significant change in functioning of the artery, as measured using FMD.

**Strengths and limitations:** In this trial 40 participants were recruited, and 39 were followed up to trial completion. A crossover design was used, with each participant receiving each supplement and a placebo in sequence. This method enabled a smaller number of participants to be used to answer the question of interest, as compared to parallel-group designs. The majority of participants in the trial were followed up. However, the trial's outcomes are surrogates for cardiovascular disease risk, measured over fairly short time periods, and no clinical outcomes were examined.

**Contribution to the evidence:** This trial adds to the evidence on the effects of nutrient supplementation on surrogate outcomes for cardiovascular disease risk. The results show that over a 6-wk study period, these surrogate outcomes are not affected by either folic acid or betaine supplementation.

## INTRODUCTION

High plasma total homocysteine concentrations may lead to cardiovascular disease [1,2], but proof that homocysteine lowering will prevent these diseases is currently lacking [3]. Some clinical trials of homocysteine lowering through B-vitamin treatment support this hypothesis [4,5], but others do not [6–10]. Several years from now, there will be data from about 50,000 patients that have been supplemented with B-vitamins or placebo [11]. However, all supplements in these trials include folic acid, which will make it impossible to distinguish between the effects of folic acid itself and the effects of homocysteine lowering per se. In an attempt to do so, we compared the effect of homocysteine lowering via folic acid supplementation and via betaine supplementation on vascular function. Betaine is involved in the remethylation of homocysteine into methionine via a different pathway than folic acid [12], and supplementation with betaine lowers plasma homocysteine in healthy volunteers to a similar extent as folic acid [13,14]. We assessed vascular function noninvasively through flow-mediated dilation (FMD) in the brachial artery [15,16]. FMD is considered a good alternative outcome measure for cardiovascular disease risk. FMD is associated with endothelial function in coronary arteries of patients [17,18]. Both coronary endothelial function [19,20] and FMD [21–25] are associated with increased mortality and morbidity risk in patients, as well as in low-risk populations. Furthermore, in a trial of anti-hypertensive treatment, those with improved FMD had a more favorable prognosis than those without improved FMD, irrespective of blood pressure lowering [26]. This supports the idea that FMD is a good surrogate marker to assess the risk of cardiovascular disease in intervention studies in low-risk populations. The study presented here shows findings from a 6-wk randomized, placebo-controlled, double-blind, crossover study in healthy men and women (50–70 y), investigating whether folic acid and betaine supplementation differentially affect FMD of the brachial artery.

## METHODS

### Participants

Participants were recruited from the pool of volunteers registered at Wageningen University in the Netherlands. Eligible volunteers were healthy as assessed by routine medical screening and a general health questionnaire; were between 50 and 70 y old; had a plasma total homocysteine concentration below 26 μmol/l; had no history of cardiovascular disease; had no hypertension; and had not used vitamin B supplements more than once a week in the 3 mo before entering the study. Out of 87 eligible participants, 40 participants (23 males) with the highest plasma total homocysteine concentrations (range, 10.2–21.7 μmol/l) were included in this placebo-controlled, double-blind, crossover study.

The study was conducted at the Division of Human Nutrition at Wageningen University (Wageningen, the Netherlands). The local medical ethics committee approved the protocol, and all volunteers gave their written informed consent.

### Interventions

Participants were randomly assigned to one out of six treatment orders, and they received each of the following supplements for 6 wk, with a 6-wk washout in between: (a) 6 g/d of betaine (BUFA B.V. Pharmaceutical Products, Uitgeest, the Netherlands); (b) 0.8 mg/d of folic acid mixed with 6 g of lactose (BUFA B.V.); (c) 6 g/d of lactose (placebo; BUFA B.V). The study supplements were dissolved in water and ingested twice per day, one half of the daily dose after breakfast and the other half after the evening meal. We used a supplementation dose of 6 g/d of betaine because we anticipated that this dose would lower fasting plasma homocysteine concentrations to a similar extent as 0.8 mg/d of folic acid [13,14,27].

### Objectives

We investigated whether lowering of fasting homocysteine concentrations, either with folic acid or with betaine supplementation, differentially affects vascular function, a surrogate marker for risk of cardiovascular disease, in healthy volunteers.

### Outcomes

FMD and plasma homocysteine concentrations were primary outcome measures. Concentrations of vitamins B_6_, B_12_, and folate in blood were secondary outcome measures.

#### FMD.

Brachial artery measurements were done in participants following an overnight fast at the end of each treatment period on days 41 and 43. We measured each participant on two separate days after each 6-wk treatment period to reduce the variation within participants. The within-participant coefficient of variation (CV = {SD/mean} × 100%) was 65% in our study, and this corresponded to previous measurements in our laboratory [28,29].

On each measurement day, participants rested on a bed for 15 min in a temperature-controlled room (20 °C −25 °C) and then we measured endothelium-dependent FMD of the brachial artery using a 7.5 MHz linear-array transducer of an ATL Ultramark 9 HDI duplex scanner (Philips Medical Systems, Bothell, Washington, United States). The measurements were done at the brachial artery of the right arm, at the site of the antecubital crease, with an inflatable cuff around the forearm. Arm and ultrasound transducer were held in position with a specially designed fixture (TAF, developed by Meijer, Vascular Imaging Center, Julius Center for Health Sciences and Primary Care, University Medical Centre Utrecht, Utrecht, the Netherlands) [29]. We chose a segment of the artery of at least 10 mm in length with clear lumen and distinctive vessel walls. All images were 10× zoomed and electronically focused. We first obtained an optimal two-dimensional B-mode ultrasound image of the brachial artery at rest and recorded three baseline images to measure baseline diameter. We then either inflated the cuff around the lower arm to a pressure of 200 mmHg or we inflated the cuff 50 mmHg above systolic blood pressure in cases in which the systolic blood pressure was >150 mmHg. The pressure was kept constant for 5 min to induce ischemia in the forearm and hand, and then the cuff was deflated and image recording was started. In the next 5 min, images of the brachial artery were frozen every 15 s. All measurements were done at the end-diastole by the use of the R-wave of the electrocardiogram. All images were recorded on super-VHS videotape for offline analysis.

The offline reading of ultrasound examinations was done using Brachial Tools, Version 3.2.6 (Medical Imaging Applications, Coralville, Iowa, United States), as has been described in detail elsewhere [29]. One reader, who was unaware of treatment allocation, read all images at the Vascular Imaging Centre of the University Medical Center Utrecht (Utrecht, the Netherlands). Each scan was read in duplicate, to limit reading variation. The coefficient of variation (CV = {SD/mean} × 100%) in calculated FMD% between readings was 22%. The reader traced the trailing edge of the adventitia–media interface at the near wall and the leading edge of the media–adventitia interface at the far wall of the brachial artery over a length of at least 3 mm. The distance between these interfaces reflects the lumen diameter. FMD was computed as the percent increase in arterial diameter: FMD% = {(maximum minus baseline)/baseline} × 100%. For reasons of clarity, we use “FMD%” as a unit of FMD measurements.

#### Blood sampling and laboratory analyses.

Venous blood was taken from the antecubital vein following an overnight fast on days 41 and 43 of each treatment period. Blood for analysis of total homocysteine was collected in vacutainer tubes containing EDTA. Samples were mixed and put on ice immediately after collection. Within 30 min, samples were centrifuged for 20 min at 2000 × *g* at 4 °C. For analyses of vitamins B_12_ and folate, blood was collected in vacutainer tubes containing clot activator and a gel to separate serum and cells. About 30 min after collection, samples were centrifuged for 15 min at 2000 × *g* at 4 °C. For analysis of vitamin B_6_, blood was collected in lithium–heparin vacutainer tubes. All samples were stored below −70 °C. Samples were coded to hide the identity and treatment of participants. All samples obtained from one participant were analyzed in the same run.

Total homocysteine concentrations (the sum of all oxidized and reduced forms of homocysteine) were measured by high performance liquid chromatography with fluorescence detection [30]. Serum folate and vitamin B_12_ were measured using a commercial chemiluminescent immunoassay system (IMMULITE 2000, Diagnostic Products Corporation, Los Angeles, California, United States) [31]. The determination of vitamin B_6_ as pyridoxal-5′-phosphate in whole blood was performed with an high performance liquid chromatography technique [32], using precolumn derivatization with semicarbazide to obtain pyridoxal-5′-phosphate–semicarbazone [33].

#### Standardization procedures.

During the study, participants were not allowed to consume supplements containing B-vitamins, antioxidant vitamins (A, beta-carotene, C, and E), or n-3 fatty acids/fish oil supplements, and participants were instructed to maintain their physical activity level, dietary habits, and smoking habits during the study.

Participants ate their self-selected diets, except on the days before blood was sampled and FMD measurements were performed (i.e., days 40 and 42 of each supplement period). On these days, we provided the participants with a standardized breakfast, lunch, dinner, and snacks. The foods consisted of normal food products, but foods rich in protein, folic acid, betaine, or choline were avoided. Participants were not allowed to consume any of their own foods during these days, except for coffee and tea. On the first day that participants received the standardized foods, they could choose the amounts they wanted to eat of the foods we provided and of coffee and tea. On all following standardized days, participants received the standardized foods in amounts similar to what they had consumed on the first day and were instructed to consume the same amounts of coffee and tea as they did on the first day. Participants were instructed to eat everything that we provided and not to eat anything else on that day. Participants prepared and ate the foods at home. Coffee and tea consumption and smoking were not allowed after 6 p.m., and dinner had to be consumed before 8 p.m.

From 10 p.m. until after the measurements the next morning (blood sampling and FMD), participants were not allowed to smoke, eat, or drink, except for water. After the measurements, a breakfast without restrictions was provided.

### Sample Size

We calculated that 30 participants would be required to detect an absolute difference of 2 FMD% relative to placebo (power = 0.8, α = 0.05). We included 40 participants in our study, anticipating that some participants might withdraw.

### Randomization and Blinding

A person not further involved in the study assigned codes to the study treatments, randomly allocated the selected participants to one out of six treatment orders, and kept the key in a sealed envelope. The participants and all others involved in this study were unaware of treatment allocation. The principal investigator performed unblinding of the treatment allocation only after the study had ended and laboratory analyses were complete.

Betaine has a bitter taste, whereas lactose is sweet. To avoid unblinding of the study by distinct differences in taste between supplements, 2 mg of quinine (chinine hydrochloridum; BUFA B.V. Pharmaceutical Products) was added per 6 g of each supplement.

### Statistical Methods

For each treatment period and for each participant, we first averaged the duplicate readings of FMD measured on day 41 and day 43 and then averaged these mean readings of days 41 and 43. For concentrations of homocysteine and B-vitamins, we averaged values of days 41 and 43 for each participant in each treatment period. Data were analyzed by the linear mixed effects models procedure in SPSS (Version 12.0). Tukey's procedure was used for pairwise comparisons and for the calculation of 95% confidence intervals (CI) between treatments. Carryover effects were checked by introducing a treatment-by-period interaction term in the model. All statistical analyses were performed with SPSS, Version 12.0.

## RESULTS

### Participant Flow and Recruitment

**Figure 1:**
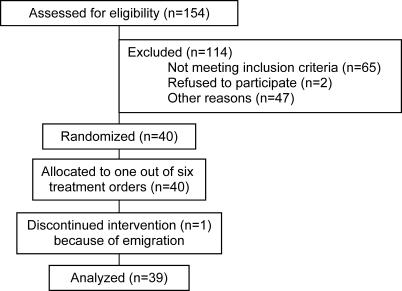
CONSORT Flowchart Participant Flow and Recruitment

Participant flow is shown in Figure 1. Volunteers were recruited from June to September 2002. The intervention started October 2002 and was completed in June 2003.

### Baseline Data

Participant characteristics at screening are shown in Table 1.

**Table 1:**
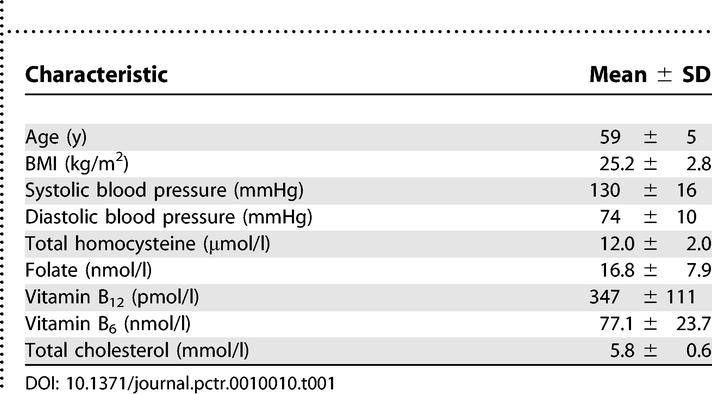
Participant Characteristics at Screening (*n* = 40)

### Numbers Analyzed

Of the 40 initial participants, 39 completed the study. One male participant withdrew from the study because he moved to another country.

### Outcomes and Estimation

Folic acid supplementation lowered fasting concentrations of homocysteine by 20% (−2.0 μmol/l, 95%CI: −2.3; −1.6) relative to placebo (Table 2). Betaine supplementation lowered fasting concentrations of homocysteine by 12% (−1.2 μmol/l, −1.6; −0.8) relative to placebo.

**Table 2:**
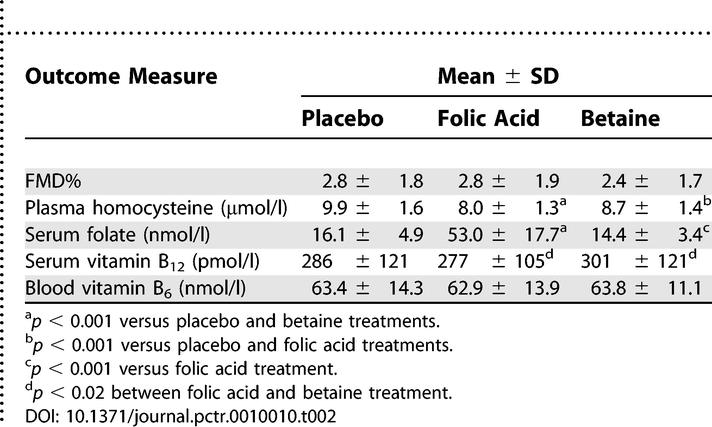
FMD%, Concentrations of Total Plasma Homocysteine, and Concentrations of B-Vitamins in 39 Participants Who Ingested Placebo, 0.8 mg/d of Folic Acid, and 6 g/d of Betaine in Random Order for 6 Wk Each, in Crossover Design

**Figure 2:**
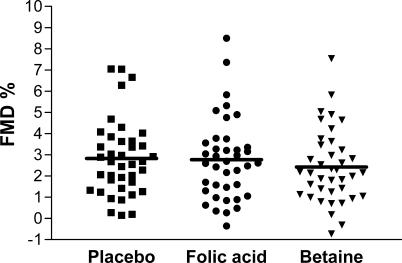
Individual Values of FMD (FMD%) of 39 Participants Who Ingested Placebo, 0.8 mg/d of Folic Acid, and 6 g/d of Betaine in Random Order for 6 Wk Each Each spot represents the mean of duplicate FMD measurements at the end of the treatment period in one participant; the line represents the mean FMD for each treatment.

FMD was not affected by supplementation with folic acid or betaine relative to placebo (Table 2; Figure 2). Relative to placebo treatment, the mean difference in FMD was only −0.1 FMD% (95%CI, −0.9; 0.7) after folic acid treatment and −0.4 FMD% (−1.2; 0.4) after betaine treatment. In addition, the mean (± SD) fasting baseline diameter of the brachial artery following placebo, folic acid, and betaine supplementation was 4.33 ± 0.62 mm, 4.31 ± 0.60 mm, and 4.44 ± 0.64 mm, respectively. Neither the baseline diameter nor the maximum diameter was affected by folic acid or betaine treatment relative to placebo.

As expected, concentrations of serum folate were increased more than 2-fold after folic acid treatment relative to concentrations after placebo treatment (+37 nmol/l, 32; 42) and after betaine treatment (+39 nmol/l, 34; 44) (Table 2). This also indicates good compliance to intake of the supplements. Concentrations of vitamins B_12_ and B_6_ did not change during folic acid supplementation relative to placebo. Betaine supplementation did not affect serum folate concentrations or concentrations of vitamins B_12_ and B_6_ relative to placebo. However, serum concentration of vitamin B_12_ was slightly higher (+24 pmol/l, 4; 44) after betaine supplementation than after folic acid supplementation. The treatment-by-period interactions test indicated that there were no important carryover effects present (data not shown).

### Adverse Events

No serious adverse events were reported in this study. Nonserious adverse events occurred 112 times in 31 participants. The adverse events were diverse and unlikely related to the treatment. Most commonly occurring events were headache/migraine (59 times) and common cold/influenza (29 times).

## DISCUSSION

### Interpretation

In our study, homocysteine lowering via folic acid and via betaine supplementation both failed to affect FMD in healthy elderly volunteers. This suggests that homocysteine, if involved in pathogenesis of cardiovascular disease, does not exert its action through a mechanism related to vascular function in healthy individuals. This is the first study to our knowledge that has compared the effects of two different homocysteine-lowering components on FMD in order to distinguish between effects of homocysteine itself and effects of folic acid.

We are confident that we designed and performed our study well. We selected apparently healthy elderly people with slightly elevated plasma homocysteine concentrations, who are expected to have a greater response to the homocysteine-lowering interventions than randomly selected individuals.

The FMD measurement itself was performed similar to previous studies done in our laboratory. In one study, replacement of dietary saturated fatty acids by dietary trans fatty acids impaired FMD after 4 wk of intervention [34], but no effect was found of a low-fat diet versus a high-oil diet on FMD [35].

We standardized the FMD measurement to a maximum of what is possible in a free-living situation. To reduce the variation in the FMD measurement due to diet, we provided all foods the day preceding the FMD measurements, and we standardized coffee and tea consumption. Further, we standardized the timing of the last time eating and smoking on the evening before measurements. During the entire study, we also asked the volunteers to keep physical activity, smoking, and dietary patterns as usual. To limit the influence of variation in FMD between participants, we chose a crossover design in our study, so that each participant was his or her own control. In addition, we know that the within-participant variability of the FMD measurement is large [28]. Therefore, we did duplicate FMD measurements in each participant on each intervention, and we also did the reading of the videotapes for each of the FMD measurement in duplicate. Indeed, the narrow 95%CI we found for the difference in FMD indicate that the power for our study was more than sufficient. Our study was powered to detect an absolute difference of 2 FMD% between the treatments and placebo, as this effect is considered to be clinically relevant and is also used in power calculations in other studies [36–38]. If we assume that the 95%CIs in our study contain the true effect, then we can say that the beneficial effect on FMD could be maximally +0.7 FMD% for folic acid and +0.4 FMD% for betaine in our population. It is unlikely that we missed a biologically relevant effect through chance fluctuations.

### Overall Evidence and Generalizability

We are not aware of other published studies that have investigated effects of betaine supplementation on FMD. Betaine lowered plasma homocysteine as expected [13,14], but we previously reported that betaine supplementation—unlike folic acid supplementation—increased LDL cholesterol concentrations by ~11% and increased triacylglycerol concentrations by ~13% [39]. Consequently, we cannot completely exclude the possibility that these increases in blood lipids induced by betaine supplementation negatively affected FMD [40] and thereby counteracted the potential positive effect of homocysteine lowering. However, this seems unlikely, since folic acid supplementation also lowered homocysteine and did not affect FMD either.

The absence of an effect of folic acid on FMD in our study is in line with results from other studies in healthy volunteers that show that chronic folic acid supplementation does not affect FMD in healthy volunteers. Only two out of eight studies in healthy volunteers reported an improvement in FMD upon long-term folic acid supplementation; the other studies did not find an effect of supplementation with folic acid alone, or in combination with other B-vitamins, on FMD (Table 3). However, it should be noted that one of the two studies [41] that found an improvement of FMD upon folic acid supplementation reported that participants ingested folic acid just before measurement of FMD. Therefore, the improvement in FMD they found could be caused by an acute effect of folic acid on FMD and not by homocysteine-lowering per se [42–44]. However, in our other study conducted at the same time as this one, we found no effect of a single dose of folic acid on FMD following a methionine load [45]. A second study [46] does not report when participants ingested the last dose of folic acid. In our study reported here, vascular function was measured in participants after they had fasted overnight, to ensure we measured chronic rather that acute effects of the supplements. The results of the study reported here are also in line with the findings of our other study on effects of acute homocysteine lowering on FMD following methionine loading [45]. Also, our findings are in line with results from recent placebo-controlled trials that found no effect of B-vitamin supplementation on secondary prevention of cardiovascular disease [6,7,9,10].

**Table 3:**
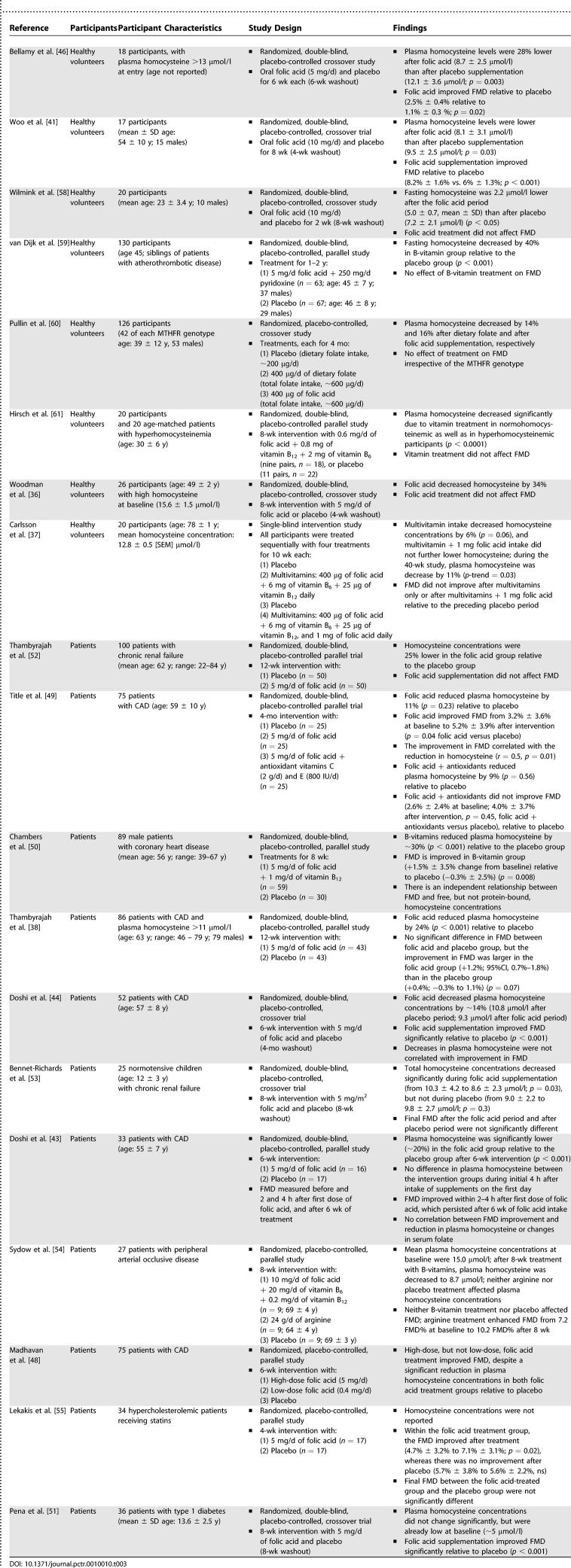
Overview of Placebo-Controlled Studies into the Effects of Folic Acid Supplementation on Vascular Function, Measured through FMD

Of course, it remains possible that homocysteine lowering affects vascular function only in patients with cardiovascular disease or participants who have other risk factors for cardiovascular disease [47]. However, data on effects of folic acid on FMD in high-risk populations are inconsistent (Table 3). Six out of 11 studies stated that FMD improved upon folic acid supplementation [43,44,48–51]. The study from Madhavan et al. [48] found an improvement in FMD only upon supplementation of high doses of folic acid (5 mg/d), but not on supplementation of low doses of folic acid (0.4 mg/d) in coronary artery disease (CAD) patients. Two studies in patients with renal failure [52,53], one study in patients with CAD [38], one study in patients with peripheral arterial disease [54], and one study in hypercholesterolemic patients receiving statins [55] found no effect of folic acid on FMD relative to placebo. Therefore, similar to the finding in healthy volunteers, studies in patients do not convincingly show that folic acid supplementation improves FMD.

### Study Limitations

Although we have performed the FMD measurement with utmost care, there are some methodological issues to discuss. First, we did not perform a nitric oxide-independent vasodilation test, usually done with sublingual nitroglycerine (NTG). The rationale for such a test is to show whether or not the artery is capable of responding to nitric oxide directly given to participants (endothelium-independent vasodilation). Since we selected healthy participants in our study, we assumed that all participants would have an FMD response, and we therefore did not confirm this with an NTG test. Moreover, treatment effects on the NTG test were not expected [34,56,57]. As we have seen an FMD response in all of our participants, we do not think that the lack of NTG measurements affects the validity of our findings.

Second, we did not perform Doppler measurements after the cuff release. The Doppler measurements reflect the stimulus (i.e., the blood flow) that elicits the FMD response, and through these measurements one may examine whether the differences between participants and between visits are due to differences in exposure to increased blood flow. We did not perform the Doppler measurements in order to optimize the measurements of the B-mode imaging. The time window to switch between B-mode and the Doppler mode in the ultrasound machine was too short to perform valid Doppler measurements and at the same time capture reliable images for lumen diameter measurements within the first period after cuff release, which is the most important phase in which dilation occurs. However, it is highly unlikely that the stimulus will differ between visits, as the measurements were done in a standardized manner, by the same technicians using an identical protocol. Moreover, the FMD was measured in duplicate on each treatment, which minimized variations in the FMD responses that were not due to the treatments. Therefore, the validity of our findings is not severely hampered by the lack of the Doppler measurement.

Finally, we added a small amount of quinine to all of the supplements in order to keep the study blinded by masking the original tastes of the supplements. Especially betaine has a somewhat distinct, unpleasant taste. Quinine is a flavoring agent, approved by the Food and Drug Administration in beverages up to 83 mg/l. We added 2 mg of quinine to each daily portion of the supplements, and we are not aware of any effects of such a small dose on vascular function. In addition, because we added quinine to all of the supplements, including placebo, it is highly unlikely that this would have influenced the results of this study.

## CONCLUSION

We showed that neither long-term folic acid nor betaine supplementation affects vascular function in healthy elderly volunteers, despite effective homocysteine lowering. This is in line with findings from other studies, most of them showing no effect of chronic folic acid supplementation on vascular function in healthy volunteers. This may indicate that homocysteine is not causally related to cardiovascular disease, but leaves open the possibility that homocysteine affects cardiovascular disease risk through mechanisms other than impaired vascular function.

## SUPPORTING INFORMATION

CONSORT Checklist Click here for additional data file. (53 KB DOC)

Trial Protocol Click here for additional data file. (115 KB DOC)

Alternative Language Abstract Click here for additional data file. Translation of the abstract into Dutch by Margreet R. Olthof.
(28 KB DOC)

## Abbreviations

CAD: coronary artery disease
CI: confidence interval
CV: coefficient of variation
FMD: flow-mediated dilation
NTG: nitroglycerine
