# Supplementary material for: Effect of Folic Acid and Betaine Supplementation on Flow-Mediated Dilation: A Randomized, Controlled Study in Healthy Volunteers
Source: PLoS Clin Trials. 2006 Jun 9;1(2):e10. doi: 10.1371/journal.pctr.0010010 (PMC1488898; doi:10.1371/journal.pctr.0010010)
Supplement: Alternative Language Abstract [file pctr.0010010.sd003.doc]

### **Abstract S1.** Translation of the abstract into Dutch by Margreet R. Olthof

### **Effect van foliumzuur en betaïne suppletie op vaatwandfunctie: een gerandomiseerde gecontroleerde interventiestudie bij gezonde ouderen**

Margreet R. Olthof, PhD; Michiel L. Bots, MD, PhD; Martijn B. Katan, PhD; Petra Verhoef, PhD

**Doel**

Wij hebben onderzocht of het verlagen van de concentraties nuchter homocysteïne via foliumzuursuppletie en via betaïnesuppletie de vaatwandfunctie beïnvloedt. Vaatwandfunctie is een surrogaat marker voor het risico van hart-en vaatziekten in gezonde vrijwilligers. Tot op heden is het onduidelijk of een hoge concentratie homocysteïne zelf, of een lage folaatstatus - de belangrijkste determinant van hoge homocysteïne concentraties - betrokken is bij de ontwikkeling van hart-en vaatziekten. Om deze reden hebben wij deze studie uitgevoerd.

**Studieopzet**

Gerandomiseerde, placebo-gecontroleerde, dubbel-blinde, crossover studie.

**Plaats**

Wageningen Universiteit, Wageningen, Nederland

**Deelnemers**

Negenendertig gezonde mannen en vrouwen tussen 50 en 70 jaar.

**Interventies**

Deelnemers namen 0.8 mg/d foliumzuur, 6 g betaïne en placebo in gedurende 6 weken elk; met 6 weken uitwasperiode ertussen.

**Uitkomstmaten**

Aan het eind van elke suppletieperiode werden plasma homocysteïne concentraties en ‘flow mediated vasodilation’ (FMD), ofwel vaatverwijding door toegenomen bloeddoorstroming, gemeten op 2 verschillende dagen.

**Resultaten**

Foliumzuursuppletie verlaagde nuchtere homocysteïne concentraties met 20% (-2.0 μmol/L, 95% betrouwbaarheidsinterval: -2.3; -1.6) en betaïnesuppletie verlaagde nuchtere homocysteïne concentraties met 12% (-1.2 μmol/L: -1.6; -0.8) ten opzichte van placebo.

Gemiddelde (±SD) nuchtere FMD na placebosuppletie was 2.8 (± 1.8) FMD%.

Suppletie met betaïne of foliumzuur hadden geen effect op FMD ten opzichte van placebo; de verschillen met placebo waren respectievelijk -0.4 FMD% (95% betrouwbaarheidsinterval: -1.2; 0.4) en -0.1 FMD% (-0.9; 0.7).

**Conclusie**

Foliumzuur- en betaïnesuppletie verbeterden vasculaire functie niet in gezonde vrijwilligers, ondanks duidelijke homocysteïneverlaging. Dit komt overeen met andere studies in gezonde vrijwilligers waarvan de meerderheid ook geen verbetering vindt in vasculaire functie na foliumzuursuppletie. Echter homocysteïne of folaat zouden mogelijk wel via andere mechanismen dan vaatwandfunctie het risico van hart-en vaatziekten kunnen beïnvloeden.
